# Case report: An ultrasound-based approach as an easy tool to evaluate hormone receptor-positive HER-2-negative breast cancer in advanced/metastatic settings: preliminary data of the Plus-ENDO study

**DOI:** 10.3389/fonc.2024.1295772

**Published:** 2024-04-16

**Authors:** Liliana Montella, Luigi Di Marino, Maria Adele Marino, Vittorio Riccio, Nunzio Del Gaudio, Lucia Altucci, Massimiliano Berretta, Gaetano Facchini

**Affiliations:** ^1^ Oncology Operative Unit, “Santa Maria delle Grazie” Hospital, ASL Napoli 2 NORD, Pozzuoli, Italy; ^2^ Breast Unit, Clinica Pineta Grande, Castel Volturno, Italy; ^3^ Oncology Operative Unit, “S.Maria della Pietà” Hospital, Casoria, Italy; ^4^ Ospedale S.Maria della Pietà, Casoria, Italy; ^5^ Department of Precision Medicine, “Luigi Vanvitelli” University of Campania, Napoli, Italy; ^6^ Molecular Biology and Genetics Research Institute, Biogem, Ariano Irpino, Italy; ^7^ Department of Clinical and Experimental Medicine, University of Messina, Messina, Italy

**Keywords:** breast cancer, hormone-responsive, radiology, ultrasound, CDK4/6 inhibitors

## Abstract

**Background:**

Hormone receptor-positive tumors are unlikely to exhibit a complete pathological tumor response. The association of CDK 4/6 inhibitor plus hormone therapy has changed this perspective.

**Case presentation:**

In this study, we retrospectively reviewed the charts of patients with a diagnosis of luminal A/B advanced/metastatic tumors treated with a CDK 4/6 inhibitor-based therapy. In this part of the study, we present clinical and ultrasound evaluation. Eight female patients were considered eligible for the study aims. Three complete and five partial responses were reported, including a clinical tumor response of 50% or more in five out of nine assessed lesions (55%). All patients showed a response on ultrasound. The mean lesion size measured by ultrasound was 27.1 ± 15.02 mm (range, 6–47 mm) at the baseline; 16.08 ± 14.6 mm (range, 0–40 mm) after 4 months (T1); and 11.7 ± 12.9 mm (range, 0–30 mm) at the 6 months follow-up (T2). Two patients underwent surgery. The radiological complete response found confirmation in a pathological complete response, while the partial response matched a moderate residual disease.

**Conclusion:**

The evaluation of breast cancer by ultrasound is basically informative of response and may be an easy and practical tool to monitor advanced tumors, especially in advanced/unfit patients who are reluctant to invasive exams.

## 1 Introduction

Advanced breast cancer includes, according to the 5th ESO-ESMO international consensus guidelines for advanced breast cancer (ABC 5) (1), both inoperable locally advanced breast cancer (LABC) and metastatic breast cancer (MBC). LABC is often considered a candidate for therapies with the aim of tumor shrinkage before the surgery, decreasing the rate of mastectomy in favor of less demolition surgery, testing the *in vivo* drug sensitivity, and tailoring treatment according to the pathological response obtained.

The indication for neoadjuvant chemotherapy in hormone receptor-positive (HR+)/HER2-negative tumors is not straightforward (2). While recent trials reach 60% of pathological response rate (pCR) in HER2-positive and triple-negative tumors and are rapidly changing therapeutic algorithms (3, 4), standard chemotherapy produces an inadequate pCR (0%–18%) (1) and breast-conserving surgery (5) approximately 60% in advanced HR+ tumors with a low Ki-67 proliferation rate. A large study starting from 134,574 HR+ breast cancer patients extracted data on 29,250 patients undergoing neoadjuvant chemotherapy and reported approximately 8% of pCR (6). As in the HER-2 and TN tumors, pCR correlates with improved survival (7).

In 2013, the St. Gallen International Expert Consensus defined luminal A and luminal B tumors as ER-positive, HER2-negative tumors having respectively Ki-67 low and PR high, or Ki-67 high or PR (8). Almost uniformly, a Ki-67 <14% is associated with a luminal A profile, while above 14% corresponds to a luminal B tumor (9). Given the fluctuating Ki-67 value across the different studies, the definition of luminal A- or B-like is often adopted. The St. Gallen International Consensus Guidelines for the treatment of early breast cancer 2021 (10), acknowledging the conclusions from another working group (9), identified the groups of tumors with Ki-67 <5% and with Ki-67 >30% and recommended chemotherapy only in the latter group. The definition of the best treatment for the in-between band remains a controversial matter. Molecular characterization of the different histological subtypes has increasingly contributed to defining precision oncology-guided therapeutic algorithms (10). Some features such as luminal B subtype, high proliferation, and lack of progesterone receptor (2) may be predictive of increased pCR rates; however, information on efficacy outcomes is scarce, while surrogate endpoints such as Ki67 reduction are often adopted in clinical trials (11).

Alternative strategies have been explored including, very recently, the combination of chemo- and immunotherapy, which lead to an increased rate of pCR in luminal-B like BC defined as HR-positive/HER2-negative, Ki-67 ≥ 20%, and/or histological grade 3 (12). In other instances, a genomic-based approach was used to differentiate high- to low-risk luminal A BC patients and address the first to neoadjuvant chemotherapy and the others to endocrine therapy (13).

The interest in endocrine preoperative strategies for HR+ positive tumors has been progressively growing. Different CDKs have been investigated in BC. They have a prognostic role and are a pharmacological target for therapeutic intervention (14).

Seven studies have investigated CDK4/6 inhibitors in neoadjuvant HR+ positive, HER-2 negative BC (**Supplementary Table S1**). Most of the studies previously reported focused on the antiproliferative effect detected *in vivo* and showed moderate clinical activity. The need for periodical biopsies to assess tumor changes for study purposes has raised some ethical issues.

To date, many studies focus on pCR as a primary objective, which is quite disappointing (15), while there is a relative lack of information coming from radiological tools used during follow-up, which could be preliminary informative and maybe predictive of treatment response. The imaging of the breast is essential in the diagnosis, staging, and monitoring of breast cancer. In clinical practice, the response is mostly evaluated by ultrasound (US) or magnetic resonance imaging (MRI), the second one showing high sensitivity in the detection of a residual tumor. Additionally, 18F-fluorodeoxyglucose positron emission tomography/computed tomography (18F-FDG PET/CT) provides functional information at baseline and after treatment and was shown to correlate with pCR (16, 17). Compared to MRI, US can be considered easier to perform, does not require the injection of exogenous contrast agents, and is less expensive. It is classically considered operator dependent, which is a limit that may be overcome by the selection of a unique and skilled operator. US is accurate in size evaluation, but less comprehensive than mammography in providing an overall view. This technique can simply be used to monitor treatment response. However, the US is not used for RECIST due to operator dependency.

We argue that an integrative approach including clinical and radiological information should best define the therapeutic algorithms of HR+, HER-negative breast cancer patients undergoing medical treatments. Therefore, we evaluate a series of advanced/metastatic HR+ patients treated with CDK 4/6 I+AI in a real-world setting, making the radiological assessment the cornerstone in comparison with clinical outcomes and pathological findings.

## 2 Case description

We retrospectively evaluated our archive of advanced and metastatic pre-and post-menopausal breast cancer patients with a pathologically confirmed ER+ and HER2− (0 or 1+ by IHC or FISH negative) invasive breast cancer treated with a combination of CDK 4/6 I+AI as per clinical practice. In detail, CDK 4/6 inhibitors used in this study were palbociclib and ribociclib. Palbociclib was used at a dose of 125 mg taken by mouth with food on 21 days and 7 days off schedule (on days 1–21 of each 28-day cycle). Ribociclib was administered at 600 mg/day: 3 weeks ON and 1 week OFF. Letrozole was the first-line endocrine treatment for all patients. Letrozole was administered every day of each 28-day cycle at a dose of 2.5 mg. Goserelin was administered to pre-menopausal subjects as a subcutaneous injection every 28 days at a dose of 3.6 mg.

No limit was formally defined for Ki-67. At baseline, an advanced stage was required related to the tumor (≥ T3 or surgically unresectable) or lymph nodes (≥ N2, upper arm edema due to breast and lymphnodes involvement). Metastatic patients at presentation with evaluable primary tumors were also considered eligible. Additional eligibility criteria included Eastern Cooperative Oncology Group (ECOG) Performance Status (PS) 0–2, adequate organ, and marrow function. Exclusion criteria included previous treatment with CDK4/6 inhibitors, organ failure, and treatment intolerance.

The study was approved by our Institutional Review Board (Campania Centro Ethical Committee approval n.391, N.Reg 22/2022) and followed the Declaration of Helsinki and Good Clinical Practice guidelines. Written informed consent was required before study entry.

The primary study aim was the evaluation of clinical and US tumor response.

Tumor and lymph nodes were clinically evaluated after 1 month of treatment and thereafter every 2 months/3 months with clinical and US examination. RX mammography, breast MRI, and PET/CT with FDG were performed after 4 months/6 months and in case of suspected progression. Computed tomography was performed as a staging procedure at study entry, after 4 months/6 months, and as clinically required.

Clinical response rate (cRR) was evaluated clinically using bi-dimensional clinical measurements and radiologically using the US before the start of treatment and every 4 months subsequently.

A single lesion, even in the case of multifocal, was chosen and evaluated at time 0, at time 1 (T1) after 4 months/6 months of therapy, and at time 2 (T2) after 8 months/12 months. B-mode US was performed in a supine and lateral position, with both upper limbs facing upwards. To study the breast lesion, a conventional B-mode US and Color-Doppler US were performed with a 4–14-MHz linear array transducer. In compliance with the 5th edition of the BI-RADS classification (18), the imaging characteristics have been recorded on a spreadsheet Excel database: size, position, shape, orientation to the skin, margins, echogenicity pattern, posterior characteristics, and vascularization. The secondary aims were the rate of response by MRI and PET, the rate of conservative surgery, the pCR obtained, changes in Ki-67, and immunohistochemistry from baseline in response to therapy and safety. The pathology exam of the resected specimen at the time of surgery was evaluated and assessed by a well-qualified pathologist. pCR was defined as the absence of invasive disease in the breast and sampled axillary lymph nodes (ypT0 ypN0). Residual tumor was defined according to the Residual Cancer Burden Index (19).

Clinically responder patients remained on treatment until disease progression if metastatic and without a fixed end-of-treatment point in case of locally advanced disease. A complete metabolic response was considered mandatory for surgical referral. If a surgical intervention was considered appropriate by the Multidisciplinary Teams, CDK 4/6 inhibitor was stopped at least 2 weeks before surgery, whereas letrozole was continued till the day of surgery.

The patients were regularly monitored with complete blood count and blood chemistry. All toxicities encountered during the study were evaluated according to the National Cancer Institute-Common Terminology Criteria for Adverse Events, v3.0. Dosage reductions were performed according to the manufacturer’s datasheet.

From a screening of 24 patients treated with CDK 4/6 I+IA, we selected eight patients with *in situ* primary breast tumors and advanced or metastatic disease having complete clinical and radiological records. All but two patients were postmenopausal (age range, 48–84 years; mean, 64 years). Two patients had a bilateral BC, three patients had advanced BC, and five had metastatic BC at study entry. The comprehensive characteristics of the patients are reported in **Table 1**.

**Table 1:** Patients’ characteristics.

Patient	Advanced/metastatic disease	Carcinoma type	Luminal	Stage	US response	Surgery	RCB
1	M	D	B	IIIC	PR	Y	3
2	A	NOS	A	II		Y	0
					CR		
3	A	L	B	IIIA	PR	N	–
						
4	M	D+D	A+B	IV	CR	N	–
5	M	L+L	B+B	IV	PR	N	–
6	M	D	A	IV	PR	N	–
7	M	D	B	IV	PR	N	–
8	A	L	A	IIIB	CR	N	–
						

*A, advanced; M, metastatic; D, ductal; L, lobular; NOS, not otherwise specified; RCB, residual cancer burden; Y, yes; N, no; CR, complete response; PR, partial response.

Two patients underwent surgery. The surgical intervention was in both cases radical mastectomy with axillary lymph node dissection.

The median number of CDK4/6 I + AI cycles before surgery was 8 (range, 6–10). The interval between the start-up of treatment and surgical procedure was 1 year and 6 months, respectively.

Before the treatment, the patients had clinically and radiologically large tumors [four out of 10 tumors were staged T2 (40%), three tumors were T3 (30%), and three were T4 (30%)]. All patients but two had clinical/radiological suspected nodal metastases.

**Figure 1:**
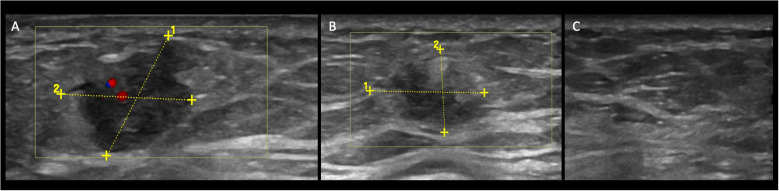
Case patient no. 4. **(A)** Color-Doppler US demonstrates a hypoechoic mass with an irregular shape and not-circumscribed margin, vertical orientation posterior, and internal vascularization at baseline. **(B)** At T1, the lesion changed the size (14 mm to 11 mm), and the vascularization at Color Doppler is now absent. The echo pattern remained hypoechoic. At T2 **(C)**, the lesion is not visible anymore.

Nine lesions having complete data were evaluated. Mean lesion size was 27.1 ± 15.02 mm [range, 6–47 mm) at the baseline; 16.08 ± 14.6 mm (range, 0–40 mm) after 4 months (T1), and 11.7 ± 12.9 mm (range, 0–30 mm) at 6 months follow-up (T2). In **Table 2**, the lesions’ size for each patient is listed. Imaging features were noted according to the Breast Imaging Report and Data System (BI-RADS) lexicon. Imaging features were noted according to the Breast Imaging Report and Data System (BI-RADS) lexicon as reported in **Table 3**. From baseline to T1, two out of seven (28%) lesions changed their shape from irregular to oval. None of the lesions changed their orientation with reference to the skin or margins. The only lesion described as complex at T1 had a combined posterior pattern at baseline. Furthermore, compared to the baseline, two lesions showed a better-defined duct infiltration after the first cycle of treatment. All lesions at T1 demonstrated absent vascularization. At T2, four lesions completely disappeared. Of the five visible lesions in the US, only 2/5 (40%) kept an irregular shape. Furthermore, all the lesions visible were hypoechoic with associated posterior shadowing. Five out of nine assessed lesions (55%) showed a clinical tumor response of 50% or more, including 4/5 (80%) complete responses and 1/5 (20%) partial responses. Changes in tumor sizes and shapes can be seen in **Figure 1**. Analysis of the area of cancer on bi-dimensional measurement before and after treatment showed that the mean values were 27.1 ± 15.02 mm at baseline, 16.08 ± 14.6 mm at T1, and 11.7 ± 12.9 mm at T2, respectively. All patients (100%%) showed a response in the US. In four cases, the lesion size halved as compared with basal.

**Table 2:** Lesion distribution and T0, T1, and T2 size by patient.

Patient	No. of lesions	Size (mm) T0	Size (mm) T1	Size (mm) T2
1	1	18	10	10
2	2	11.5	0	0
3	3	44	40	30
4	4	14	10	0
5	5	36.5	30.5	23
6	6	47	33	30
7	7	31	11	14
8	8	36	10	0
	9	6	0	0

**Table 3:** BI-RADS descriptors at T0, T1, and T2 evaluation.

BI-RADS descriptors		T0	T1	T2
Shape	Irregular	7/9 (78%)	3/7 (43%)	2/5 (40%)
	Oval	1/9 (11%)	3/7 43%)	2/5 (40%)
	Round	1/9 (11%)	1/7 (14%)	1/5 (20%)
Orientation	Parallel	4/9 (44%)	3/7 (43%)	3/5(60%)
	Not-parallel	5/9 (56%)	4/7 (57%)	2/5 (40%)
Margins	Circumscribed	–	–	–
	Not-circumscribed	9/9 (100%)	7/7 (100%)	5/5 (100%)
Echo pattern	Hypoechoic	5/9 (56%)	6/7 (86%)	5/5 (100%)
	Complex cystic and solid	3/9 (33%)	1/7 (14%)	–
	Heterogeneous	1/9 (11%)	–	–
Posterior features	None	–	–	–
	Enhancing	–	–	–
	Shadowing	5/9 (56%)	6/7 (86%)	5/5 (100%)
	Combined pattern	4/9 (44%)	1/7 (14%)	–
Associated features	Skin changes	7/9 (78%)	2/7 (28%)	2/5 (40%)
	Edema	1/9 (11%)	3/7 (43%)	1/5 (20%)
	Duct infiltration	1/9 (11%)	2/7 (28%)	2/5 (40%)
Vascularization	Absent	2/9 (22%)	7/7 (100%)	5/5 (100%)
	Vessels in rim	1/9 (11%)	–	–
	Internal	6/9 (67%)	–	–
No lesion			2/9 (22%)	5/9 (56%)

The two patients that underwent surgery were selected because of a metabolic complete response by PET/CT. In detail, one of them had a complete radiological response, the other reported a partial response. The radiological complete response found confirmation in a pCR, while the partial response matched an RCB-II (moderate residual disease). In this latter case, Ki-67 lowered from 40% to 6%.

Treatment with the CDK 4/6 inhibitors was generally well tolerated. In detail, only two patients aged more than 65 required a lower dose, and only one patient aged 84 years needed both dose reduction and prolonged drug rest to allow hematological recovery.

## 3 Discussion and conclusion

Approximately 80% of patients with breast cancer are diagnosed at an age >50, and luminal A BC is most diagnosed in over 70 years population (20). This epidemiological landscape requires customized management in real-world practice that regards the frequent old age of our patients’ population and acceptance of treatment/diagnostic procedures.

HR+ breast cancer larger than 2 cm, with involved lymph nodes and high Ki-67 index, have been considered candidates for preoperative treatments. Assessment of breast cancer treatment response is challenging, with the potential of underestimation or overestimation of residual cancer for the available different imaging techniques. Both morphological and functional imaging methods can be used to assess the response to treatments. Morphological techniques, such as full-field digital mammography (FFDM), digital breast tomosynthesis (DBT), and the US, and advanced techniques, such as breast MRI, contrast-enhanced spectral mammography (CESM), 18F-FDG PET/CT, and MRI, are nowadays pivotal in breast cancer. The selection of the imaging methods depends on the availability and should always be established in the multidisciplinary tumor board (21–25).

Ultrasound may be useful to predict the molecular subtype before pathological diagnosis (26) as reported by Zhu et al., having evaluated ultrasound features concerning more than 80 patients. This report emphasizes the value of the US in breast cancer that should not be neglected within the array of more complex radiological techniques.

In our series, the number of each histotype (**Table 1**) limited an extensive description of US reports correlated to histology.

US is superior to FFDM, DBT, and clinical breast examination in monitoring the response to NAC and is overall well tolerated by the patients. Sonographic evaluation of the residual tumor includes not only the size but also changes in tumor echogenicity and a decreased mass stiffness, and both could be considered good predictors of a pCR (27, 28). US is not used for RECIST due to operator dependency. However, US is easily accepted by patients and may be routinary and repeatedly performed. Moreover, the percentage of US diameter reduction after three cycles of treatment showed acceptable sensitivity and specificity response in a study on 64 patients (27).

In our cases, US demonstrated how the lesions changed not only in size but also in echogenicity and, by using the BI-RADS lexicon, the associated features described at the baseline. The most salient changes were the absence of vascularization after the first cycle of treatment and the changes in echogenicity after the second cycle of treatment.

Several factors may influence US breast evaluation. Body fat-driven obesity and breast fat density and their changes according to menopausal status are known factors influencing radiological assessment (29).

Efforts have been devoted to unraveling the impact of body mass index (BMI) on the therapeutic response among breast cancer patients, yet controversy persists. A recent meta-analysis disclosed that overweight/obese patients exhibited a lower pCR rate compared to under/normal weight counterparts (30).

In detail, also the assessment of the axilla may change with varying patient BMI, thus possibly conditioning the selection of a given radiological technique (31).

Defining a precise sensitivity for ultrasound alone proves challenging, with variations strongly dependent on lesion size, breast tissue type, and, as with all methods, patient selection.

While breast density stands as an independent risk factor for breast cancer, the widely acknowledged association between overweight/obesity and breast cancer development, particularly impacting the prognosis of HR-positive postmenopausal breast cancer, prompts further exploration. Various studies suggest that factors such as the chronic inflammatory state, circulating adipokines, insulin, insulin-like growth factor (IGF), and sex hormones may play pivotal roles in mediating the link between overweight/obesity and breast cancer (32).

The histological BC subtypes notoriously correlate with a different response to medical treatment. A different sensitivity in detecting pCR after NAC was found to be related to parameters such as estrogen receptor expression, on the one hand and HER-2 overexpression, luminal B, or Ki-67 proliferation >14%, on the other hand, respectively favoring MRI and PET-CT (33). Therefore, the need for radiological studies set by subtypes is progressively acknowledged (34).

While luminal A tumors are poorly chemoresponsive and better candidates for the association of CDK4/6 and aromatase inhibitors (CDK 4/6 I+AI), luminal B tumors represent an area of investigation. The NCT04137640 (35) is investigating the efficacy of the CDK 4/6 inhibitor palbociclib plus letrozole in comparison to chemotherapy in locally advanced breast cancer with ≤30% Ki-67 with group allocation according to Ki-67. The PREDIX LumB (NCT02603679) (35) is a phase II study that enrolled luminal B tumors and compares two sequences of treatments: weekly paclitaxel versus the combination of the CDK 4/6 inhibitor palbociclib and standard endocrine treatment for an initial phase of 12 weeks with following cross-over for further 12 weeks of treatment (neoadjuvant phase: 24 weeks) and adjuvant chemotherapy for both groups.

RIBOLARIS (NCT05296746) (35) used ribociclib as neoadjuvant treatment for clinically high-risk ER+ and HER2− breast cancer with a choice of CDK 4/6 inhibitor prosecution or alternately treatment with chemotherapy in the adjuvant phase according to the pathological and biological response assessed by Prosigna.

CDK 4/6 I+AI combination shows distinctive features as compared to other therapies. First, from a biological point of view, the inhibition of growth arrest may be reversible. CDK4/6 inhibition induces cytostatic effect in cell cultures and solid tumor models with potential regression associated with the intrinsic tumor cell turnover (36). Variably, according to the studied models, growth arrest may be irreversible or not (36). This aspect translates into Ki-67 changes during and at treatment, stopping with a rapid rise while off-therapy. From a clinical point of view, further consequences are hypothetical prolonged treatments to achieve the best response and redefinition of the best time for surgical intervention, which, in clinical studies, canonically falls 16–20 weeks after the start of treatment. This window may be functional for chemo- but not for CDK 4/6 inhibitor-based treatment. In line with this concept, NEOLETRIB trial (NCT05163106) (35), RIBOLARIS, and FELINE pre-specified a study treatment of 24 weeks or at least 6 months.

Ki-67 determination at 2–4 weeks of treatment has been considered the most accurate surrogate endpoint in neoadjuvant endocrine treatments. However, the recognized limits are represented by heterogeneous Ki-67 within the tumor, which required more than one biopsy to best collect comprehensive information and the invasive nature of repeated biopsies (37). From a biological approach, we then moved to a radiological-centered approach. This pattern has been typically described by MRI but is also detected by US and results to be predictive of worse outcomes compared to concentric shrinkage (38).

Similar to MRI, US is a technique that does not expose the woman to radiation and its related risks. However, opposite to MRI, there are no contraindications to the breast US. Furthermore, breast US is a valid tool that is widely available, and it is not prone to time slots and shortage of equipment as much as MRI. Furthermore, breast US is cheap, its cost being comparable to that of mammography and much lower than that of breast MRI REF. In Italy, the cost of a single breast MRI (250€) is up to seven times higher than that of one breast US (35.89 €). Yet, these ways are less expensive compared to the costs of a breast MRI in the US (>500 $).

Therefore, the US preserves its role as an easy and practical tool to monitor local response to medical treatments, especially in unfit and elderly patients, reserving a delayed time for more demanding and expansive exams. Further prospective studies including a larger number of patients should define the value of each tool and best guide diagnostic guidelines drafting in NAC. At present, we highlight the role of a basic breast US in the follow-up of advanced/metastatic BC with *in situ* tumors. In a time of limited economic resources, the appropriate use of each radiological technique should be encouraged.

## Data availability statement

The raw data supporting the conclusions of this article will be made available by the authors, without undue reservation.

## Ethics statement

The study was approved by the Campania Centro Ethical Committee. The study was conducted in accordance with the local legislation and institutional requirements. The participants provided their written informed consent to participate in this study. Written informed consent was obtained from the participant/patient(s) for the publication of this case report.

## Author contributions

LM: Writing – review & editing, Writing – original draft. LDM: Writing – review & editing, Resources, Investigation, Conceptualization. MM: Writing – review & editing, Writing – original draft, Supervision, Project administration, Formal analysis, Data curation. VR: Writing – review & editing, Visualization, Supervision. NG: Writing – review & editing, Validation, Supervision. LA: Writing – review & editing, Visualization, Validation, Supervision. MB: Writing – review & editing, Visualization, Validation, Supervision. GF: Writing – review & editing, Visualization, Validation, Supervision.
